# Adding a Second iTBS Block in 15 or 60 Min Time Interval Does Not Increase iTBS Effects on Motor Cortex Excitability and the Responder Rates

**DOI:** 10.3390/brainsci12081064

**Published:** 2022-08-11

**Authors:** Ilya Bakulin, Alfiia Zabirova, Dmitry Sinitsyn, Alexandra Poydasheva, Dmitry Lagoda, Natalia Suponeva, Michael Piradov

**Affiliations:** Research Center of Neurology, 125367 Moscow, Russia

**Keywords:** intermittent theta-burst stimulation, metaplasticity, transcranial magnetic stimulation, non-invasive brain stimulation, motor cortex excitability

## Abstract

The use of metaplasticity-based intermittent theta-burst stimulation (iTBS) protocols including several stimulation blocks could be a possible approach to increasing stimulation effectiveness. Our aim was to investigate the neurophysiological effects of two protocols with a short and a long interval between blocks. Seventeen healthy volunteers received four protocols in a pseudorandomized order: *iTBS 0-15* (two blocks of active iTBS of primary motor cortex (M1) separated by 15 min and a control stimulation block of the vertex in 60 min from the first block); *iTBS 0-60* (active iTBS, a control block in 15 min, and an active block in 60 min); *iTBS 0* (active iTBS and two control blocks with the same intervals); and *Control* (three control blocks). The motor evoked potentials (MEPs) were measured before the first and after the second and third blocks. We have shown no significant differences between the effects of the protocols on both the motor cortex excitability and the responder rates. No significant changes of MEPs were observed after all the protocols. The reliability for the responsiveness to a single block between two sessions was insignificant. Our data confirm low reproducibility of the response to iTBS and suggest that the use of repeated protocols does not increase the responder rates or neurophysiological effects of iTBS.

## 1. Introduction

Non-invasive brain stimulation techniques, particularly transcranial magnetic stimulation (TMS), have been intensively developed during recent decades [1,2]. The application of TMS for the treatment of different diseases of the nervous system is based on the ability of the protocols of repetitive stimulation to induce a long-term neuromodulation effect, which persists after the termination of stimulation and is probably based on its influence on synaptic plasticity [2,3,4].

Although there are numerous studies in the field of TMS application as a therapeutic technique in different disorders, a high variability of its effect remains a substantial limitation for TMS use in clinical practice [1,5,6,7,8,9]. High variability is also an issue with the use of a relatively new, highly effective theta-burst stimulation (TBS) protocol [10,11,12]. In particular, it has been shown that the expected neurophysiological effect of TBS on motor cortex excitability is observed in less than half of healthy volunteers [6,13,14]. According to the results of a recently published study, in which TBS effects on the motor cortex were analyzed, only ~8% of participants could be categorized as responders across two visits of intermittent TBS (iTBS) or continuous TBS (cTBS) [14].

Many different approaches to increase the effectiveness of TMS have been suggested, including personalization of the stimulation protocols, e.g., of the TMS target [15] or frequency [16,17], investigation of the predictors of stimulation effectiveness [18], development of brain-state dependent stimulation approaches [19,20,21], etc.

One of the promising directions in which to optimize therapeutic brain stimulation techniques is based on accounting for the mechanisms of metaplasticity (plasticity of synaptic plasticity). The hypothesis of metaplasticity is based on the assumption that the magnitude, direction, and length of synaptic plasticity are dependent on the previous synaptic activity [22,23,24]. Metaplasticity can reduce or even reverse the effect of synaptic plasticity (so-called homeostatic metaplasticity) or increase it (non-homeostatic or additive metaplasticity). According to the theory of bidirectional synaptic plasticity, also called the Bienenstock–Cooper–Munro (BCM) theory, a low level of previous synaptic activity favors long-term potentiation (LTP) over long-term depression (LTD), whereas a high level of previous activity favors LTD over LTP [25]. In the case of TMS application, metaplasticity mechanisms can be taken into account if several TMS protocols are combined or if other techniques are applied before or after TMS, e.g., exercise, cognitive training, psychotherapy, etc. A number of studies have convincingly shown that metaplasticity is crucial for the effects of different brain stimulation protocols, including the possibility to both increase and decrease these effects (for a detailed description see the reviews [26,27,28]). Metaplasticity might have a significant contribution to the effects of so-called accelerated stimulation protocols, which are based on the multiple repetitions of several stimulation blocks [29,30].

The effects of stimulation protocols consisting of two or more blocks depend both on the type of each separate block and the time interval between them. In line with BCM theory, a study has shown that the combination of differently directed stimulation blocks (inhibitory and facilitatory) increases the iTBS effect, probably inducing non-homeostatic plasticity, whereas the combination of two blocks of the same type might lead to homeostatic plasticity [31]. However, according to the results of other experiments, an essential factor influencing the effects of repeated (combined) protocols is the time interval between separate stimulation blocks [28,32,33,34]. In particular, different effects are described for repeated protocols including the same stimulation blocks but separated by different time intervals (reviewed by Hassanzahraee et al., 2018) [28]. Experimental studies have shown that a short (less than 15 min) interval between two interventions can reduce or even offset the stimulation effects due to the induction of homeostatic plasticity [31]. Increasing this interval to 60 min can enhance the effect of intervention by the induction of additive metaplasticity [33,35]. Based on the results of experimental studies of NIBS in healthy volunteeers, the hypothesis of a “critical time window” was proposed, stating that the application of a second stimulation block in the middle third of the expected aftereffect of the first block leads to homeostatic metaplasticity, whereas time intervals less or more than this window lead to additive metaplasticity [28]. In human studies, protocols with several NIBS blocks separated by short intervals have been applied so far. Therefore, a novel approach of our study is the investigation of the neurophysiological effect on motor cortex excitability of a repeated iTBS protocol with a long (60 min) interval between stimulation blocks in healthy humans. Moreover, we compared it to another metaplasticity-based repeated protocol with a different time interval between separate iTBS blocks, with regard to both the responder rates and the neurophysiological measures of motor cortex excitability.

The main aim of this study was to investigate the influence of repeated iTBS protocols with a short (15 min) or long (60 min) time interval between the active stimulation blocks on the variability of the neurophysiological effects of iTBS applied to the primary motor cortex (M1), as well as to assess the effects of these metaplasticity-based repeated protocols on the motor cortex excitability. Based on the idea of the “critical time window” described above [28], our primary hypothesis was that a short interval between blocks would decrease or even reverse the effects of a single block, whereas a long interval could increase the neurophysiological effects of iTBS or the responder rates by inducing additive metaplasticity processes. The effect of the repeated iTBS protocols was compared to the effects of a single iTBS block and of the control stimulation of a functionally insignificant area (vertex).

## 2. Materials and Methods

### 2.1. Participants

The study had a single-blinded randomized cross-over design and was conducted in the Research Center of Neurology (Moscow, Russia) from 2021–2022, in accordance with the Declaration of Helsinki. It was approved by the Local Ethical Committee of Research Center of Neurology (protocol No 8-8/21, 15 September 2021). All the participants had given a written informed consent before the inclusion into the study. 

*Eligibility criteria included:*

- Age between 18–40;
- Right-handedness according to the Edinburgh Handedness Inventory (EHI) [36].

Participants were *not included* in the study in case of:

- Neurological or psychiatric disorders (at the time of inclusion or a history of such medical conditions);
- Severe diseases of other systems;
- Intake of drugs with an influence on the central nervous system at the time of inclusion;
- Contraindications to TMS and/or MRI (implanted cardiac pacemaker or intracardiac catheters, electronic pumps, cochlear implants, ferromagnetic metallic clips or stents, metallic foreign bodies, implants and post-surgical devices, claustrophobia, pregnancy) [37];
- Epileptiform activity on routine EEG.

*Exclusion criteria were as follows:*

- Severe adverse events (AEs) during the TMS procedure (epileptic seizures, syncopes, and others);
- Occurrence of psychiatric or neurological disorders after the inclusion into the study;
- Occurrence or exacerbation of existing somatic diseases after the inclusion;
- Installation of cardiac pacemaker, intracardiac catheters, or brain surgery with implantation of metallic devices in the skull;
- Pregnancy;
- Participants’ refusal to participate in the study.

The design and eligibility criteria were not changed after the study’s commencement.

### 2.2. Study Protocol

A routine EEG recording (15 min, including baseline activity and eye-opening reactivity, as well as rhythmic photic stimulation and hyperventilation) was performed for all the participants before the start of TMS for safety reasons (exclusion of frequent epileptiform activity), using a Neuron-Spectrum-4/P device (Neurosoft, Ivanovo, Russia). A structural brain MRI (3D T1 multiplanar reconstruction (MPR) sequence, voxel size 1.0 × 0.977 × 0.977 mm^3^, 176 sagittal slices) was obtained for all the participants using Siemens MAGNETOM Verio or MAGNETOM Prisma MRI scanners (Siemens Healthcare GmbH, Erlangen, Germany) to create an individual 3D model of the brain for navigated TMS. 

**Figure 1:**
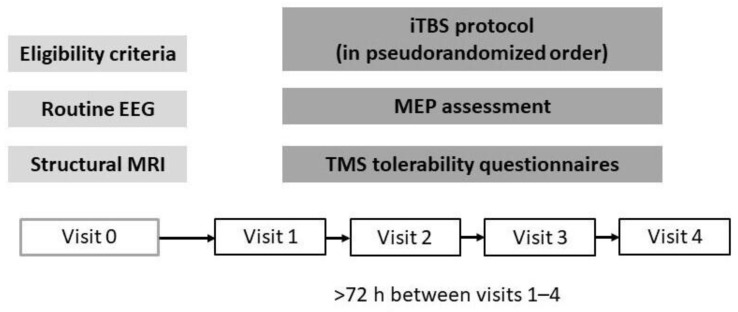
Study protocol. At the visit 0, healthy volunteers were screened for eligibility criteria, and a routine EEG as well as a structural MRI were performed. Then, repeated iTBS protocols were applied in a pseudorandomized order (visits 1–4).

The study included 4 sessions of intermittent theta-burst stimulation (iTBS) of the primary motor cortex (M1) or the vertex area on separate days with a “wash-out” interval between sessions of at least 72 h in order to minimize the influence of the previous session on baseline motor cortex excitability in view of the fact that this influence has been shown to last less than 1 h after a single iTBS block [12] (Figure 1).

**Figure 2:**
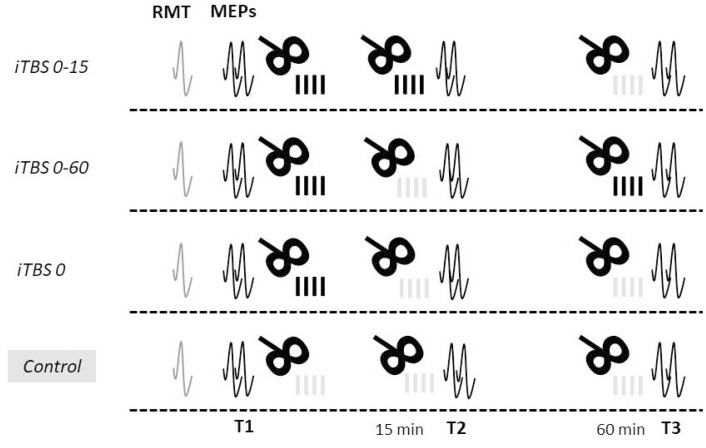
Repeated iTBS protocols and timing of the motor evoked potential (MEP) assessment at each visit. *iTBS 0-15*—two blocks of active iTBS of M1 separated by 15 min followed by iTBS of the vertex 60 min after the end of the first block; *iTBS 0-60*—an active iTBS, iTBS of the vertex 15 min after it, and a second block of active iTBS 60 min after the end of the first block; *iTBS 0*—a block of active iTBS followed by two blocks of iTBS of the vertex 15 and 60 min after the first block; *Control*—three blocks of iTBS of the vertex with the same intervals between blocks as in the other protocols.

All the participants received one session of each of the four protocols: two variants of the repeated protocol (with short and long intervals between iTBS blocks), a standard protocol, and a control protocol (Figure 2). All attempts were made to schedule all visits for a participant at the same time of day or, at least, in the same time window (9 a.m.–1 p.m. or 2 p.m.–6 p.m.). The order of the protocols for each participant was obtained by drawing a pseudorandom permutation of four numbers. The participants were blinded to the protocols that they received.

### 2.3. Transcranial Magnetic Stimulation

#### 2.3.1. iTBS

During the procedure, all the participants were comfortably seated in an adjustable chair. Navigated iTBS was performed using the MagPro X100 + MagOption (Tonica Elektronik A/S, Farum, Denmark) stimulation device with a figure-of-eight coil with a liquid cooling system. A robotic system was used for the placement of the coil (Axillum Robotics TMS-Cobot, Axillum Robotics, Schiltigheim, France) with the Localite TMS Navigator System (Localite GmbH, Bonn, Germany). For each stimulation block, a standard iTBS protocol was used: bursts, consisting of three stimuli with a frequency of 50 Hz, were applied with a frequency of 5 Hz for 2 s with an interval of 8 s between them. The total number of stimuli per block was 600. The stimulation target was localized in the M1 at the “hot spot” of the first dorsal interosseous muscle (FDI) cortical representation for active iTBS and at the vertex (functionally irrelevant area) as a control stimulation, which was used as a control condition in previous studies investigating repeated iTBS protocols (e.g., [38,39]). A robotic neuronavigation system was used to accurately determine and maintain the position of the stimulating coil.

The iTBS intensity was set at 75% of the resting motor threshold (RMT), determined according to the Rossini–Rothwell algorithm before each TMS session [40]. The choice of iTBS intensity was based on the data on the most pronounced modulating effect of TBS when using this stimulation intensity [41].

The stimulation protocols included 4 varied combinations of 3 active and control stimulation blocks, as follows (Figure 2). Time intervals between stimulation blocks were determined based on the “critical window” hypothesis proposed by Hassanzahraee et al. (2018) [28].

- Repeated iTBS protocol with a short interval between blocks (*iTBS 0-15*): two blocks of active stimulation with an interval of 15 min between them and a control stimulation block in 60 min from the end of the first block;
- Repeated protocol with a long interval between blocks (*iTBS 0-60*): a block of active stimulation was followed by a block of control stimulation with an interval of 15 min between them, and another block of active stimulation was applied in 60 min from the end of the first block;
- Standard iTBS protocol (*iTBS 0*): firstly, one block of active stimulation was applied, followed by two blocks of control stimulation in 15 and 60 min from the end of the first block;
- Control protocol (*Control*): three blocks of control stimulation were applied: at baseline, 15 min, and 60 min after the end of the first block.

#### 2.3.2. Motor Evoked Potential (MEP) Acquisition

The peak-to-peak MEP amplitude was measured to assess the stimulation effects on the motor cortex excitability before the first block of stimulation (T1), as well as after the end of the second (15 min; T2) and third (60 min; T3) blocks of stimulation. The MEPs were recorded from the FDI of the right hand. Ag–AgCl surface adhesive electrode-pairs (Neurosoft, Ivanovo, Russia) were placed on the belly and tendon of the muscle and the ground on the right radial styloid process. Suprathreshold stimulation intensity (120% of the RMT) was applied in order to register MEPs in the resting state of the target muscle. Based on a previous study investigating repeated TBS protocols, 30 MEPs were registered at each time point [42]. The first MEP was excluded from all calculations because of the possible startle reaction. The baseline activation of the hand muscles on the EMG was controlled, and all MEPs where significant activity was observed were not included and were replaced by new MEPs (up to 2 measurements in a session). The MEPs with amplitudes of less than 50 µV were considered as zero.

#### 2.3.3. Tolerability Assessment

All the participants filled in standardized questionnaires to assess the safety and tolerability of the procedure: a questionnaire regarding AEs during a stimulation session was filled 10 min after the stimulation, and a questionnaire regarding AEs occurring within 24 h after the TMS was completed before the next session. 

### 2.4. Statistical Analysis

Matlab R2017a software (MathWorks, Natick, MA, USA) was used for the statistical analysis. The effect of each iTBS protocol at time points T2 and T3 was measured by the mean MEP amplitude divided by the corresponding value at T1 and expressed in percent. Wilcoxon’s signed-rank test was used both for testing the effect of each individual protocol at T2 and T3 (the difference from the baseline value of 100%) and for comparing the effects between different pairs of protocols at each of the time points T2 and T3. We also assessed the correlations between the baseline MEP amplitudes, as well as the coefficients of variation (CVs) of the baseline MEP amplitudes, with the response to iTBS using the Spearman correlation coefficient.

Depending on the mean amplitude µ at each time point in every protocol (expressed in percent of the baseline), the subjects were classified as responders and further subdivided into facilitators (µ > 110% of the baseline) and inhibitors (µ < 90%), as well as non-responders (90% < µ < 110%). The rates of each type of response (facilitation and inhibition) were compared between different protocols and time points using the exact binomial test. The obtained responder classifications were also tested for pairwise associations between different conditions using the Fisher’s exact test. 

The above analyses were repeated with the exclusion of the MEP measurements with the largest within-session variability in order to reduce random errors in the stimulation effects, which may decrease the power of the group tests. To this end, we selected 80% of the measurements with the lowest coefficients of variation (CVs) of the individual MEP amplitudes. Each analysis (concerning one or two protocols and one or two time points) was performed on the subset of subjects in which the required measurements were selected (the value at T1 was required in all cases for normalization). Similar analyses were also performed with the exclusion of the subjects with the largest baseline MEP variability between sessions. For this, we selected 50% of the subjects having the lowest CVs of the mean MEPs at T1 in all the four sessions.

Additionally, two reliability analyses for the effects of this block combination were performed, one using the Spearman correlation of normalized mean amplitudes at T2 between the protocols *iTBS 0-60* and *iTBS 0*, and the other one testing for the association between facilitation at T2 in the protocols *iTBS 0-60* and *iTBS 0* by the Fisher’s exact test (and similarly for inhibition). The Bonferroni correction was applied to sets of comparisons of the same type.

Subjects having fewer than half nonzero MEPs in one or more baseline measurement sessions in the four protocols were excluded due to the unreliable estimation of the mean MEPs in such cases.

## 3. Results

### 3.1. Study Sample

In total, 22 healthy volunteers were included in the study, among them two participants had not completed all the study protocols due to logistic reasons, and two participants refused to continue iTBS because of AEs (headache within 24 h after a session and an unpleasant feeling of pressure at the stimulation site). Thus, 18 volunteers completed the study (8 males, mean age 27.4 y.o.). The data of one participant were removed from the analysis because of less than half nonzero MEPs (MEPs with amplitudes of 50 µV or more) were registered in the baseline of a session.

### 3.2. Effects of Single Protocols

The baseline mean MEP amplitudes in the four protocols had sample distributions with the following characteristics: *iTBS 0-15*: range 135 to 1005 μV, median 490 μV; *iTBS 0-60*: range 157 to 1131 μV, median 498 μV; *iTBS 0*: range 146 to 1232 μV, median 510 μV; *Control:* range 141 to 1418 μV, median 484 μV. For individual plots, see Supplementary file (Figures S1–S4). The MEP amplitudes were not significantly different between the protocols: Friedman’s test, *p_unc_* = 0.37, χ^2^(3) = 3.14.

**Figure 3:**
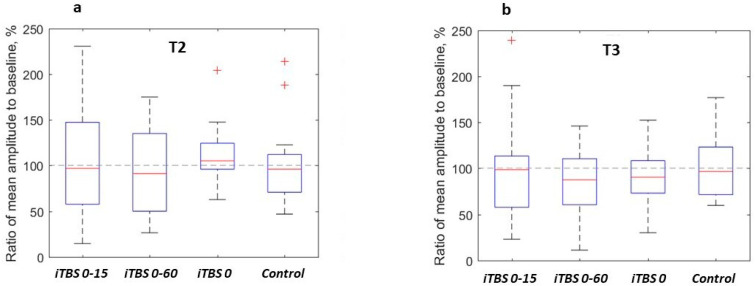
Effects of the four stimulation protocols on MEP amplitudes ((**a**)—at time point T2, (**b**)—at T3). For each protocol in every subject, the mean MEP amplitudes at time points T2 and T3 were divided by the mean amplitude at T1 and expressed in percent. The central mark indicates the median, the box indicates the quartiles, and the whiskers extend to the most extreme data points not considered outliers. Outlier values are defined as lying above the upper quartile or below the lower quartile by more than 1.5 interquartile range and marked with ‘+’ symbols. The horizontal dashed line indicates 100% (baseline).

In the four studied protocols, the mean MEP amplitudes at T2 and T3 in the different subjects ranged from 11% to 239% of the baseline mean MEP (Figure 3, Table 1). In the comparisons with the baseline (100%), no protocol showed *p_unc_* < 0.05 at either time point (Wilcoxon’s signed-rank test), with *iTBS 0* at T2 yielding the lowest uncorrected *p*-value equal to 0.1 (median effect 105%). We compared the effects of each protocol at T2 and T3, and the *iTBS 0* had *p_unc_* = 0.01 (larger MEPs at T2 than T3, median of the differences 14.1% of the baseline), while the other protocols gave *p_unc_* > 0.05. After correction for multiple comparisons, all the differences became non-significant (in *iTBS 0, p_corr T2-T3_* = 0.12).

There was no association between the mean baseline MEP amplitude and the response to iTBS (expressed in percent of the baseline) in any of the four protocols at T2 or T3 (*p_unc_* > 0.05, Spearman correlation). 

Tests of the association between the coefficients of variation of the MEP amplitude at the baseline with the response to iTBS were performed for all the four protocols at T2 and T3 and yielded *p_unc_* < 0.05 only for the control protocol at T2 (Spearman’s ρ = −0.5, *p_unc_* = 0.04), not passing a Bonferroni correction (*p_corr_* = 0.32). Of note, all the eight sample Spearman correlation coefficients were negative.

### 3.3. Comparisons of the Effects of Protocols

The effects of different protocols were compared using Wilcoxon’s signed-rank test. This yielded *p_unc_* > 0.05 in the pairwise comparisons of the MEPs at T2 and T3 of all the protocols to each other (Table 2).

### 3.4. Comparisons of Responder Rates

**Figure 4:**
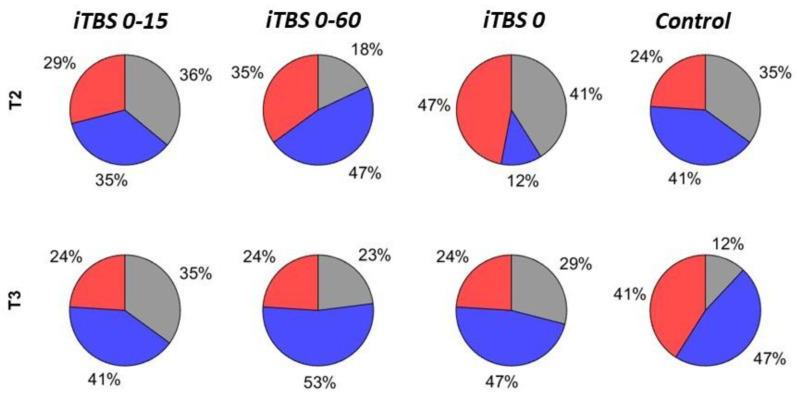
Pie charts of percentage of facilitators (red, mean amplitude µ ≥ 110% of the baseline), inhibitors (blue, µ ≤ 90%), and non-responders (grey, 90% < µ < 110%).

Depending on the mean amplitude µ at each time point in every protocol (expressed in percent of baseline), the subjects were classified as responders and further subdivided into facilitators (µ > 110% of the baseline) and inhibitors (µ < 90%), as well as non-responders (90% < µ < 110%). The proportions of the subjects belonging to each category are shown in Figure 4. The rate of facilitation ranged from 24% to 47%, while the proportion of inhibitors was between 12% and 53%. 

The rates were compared between different protocols and time points using the exact binomial test. There were no results with *p_unc_* < 0.05 in the comparisons of the facilitation rates in the different protocols (Table 2). The inhibition rate at T2 was lower in the protocol *iTBS 0* (12%) than in *iTBS 0-60* (47%, *p_unc_* = 0.03) (Table 2). In the comparisons of the responder rates in the same protocol at different time points, results with *p_unc_* < 0.05 were obtained only for the inhibition rate at T2 in *iTBS 0* (12%), which was lower than the inhibition rate at T3 in the same protocol (47%, *p_unc_* = 0.03) (Table 3). However, these differences became non-significant when *p* was corrected for multiple comparisons by applying the Bonferroni correction (*p_corr_* = 0.36 for inhibitor rate at T2 in *iTBS 0* and *iTBS 0-60*, as well as for the inhibitor rates at T2 and T3 in *iTBS 0*).

### 3.5. Correlations of Responder Classifications

The responder classifications in all the conditions were tested for pairwise associations using the Fisher’s exact test (Table 4). With regard to the facilitation at different time points in the same protocol, these tests yielded *p_unc_* < 0.05 for the correlations between T2 and T3 in the protocol *iTBS 0-60* (*p_unc_* = 0.006). When the correlations of the responses to the different protocols were calculated, a significant correlation was observed between the facilitation at T3 in *iTBS 0-15* and *iTBS 0-60* (*p_unc_* = 0.02). The correlations were positive, i.e., the facilitation in one condition increased the probability of facilitation in the other. The tests of the association of inhibition in different conditions produced *p* < 0.05 for the positive correlations between T2 and T3 in the *iTBS 0-15* (*p_unc_* = 0.04) and between T2 and T3 in the *iTBS 0-60* (*p_unc_* = 0.0004). After correcting for multiple comparisons (32 tests), only the correlation between the inhibition at T2 and T3 for *iTBS 0-60* remained significant (*p_corr_* = 0.013).

### 3.6. Analysis Excluding the MEP Measurements with Largest within-Session Variability

The above analyses were repeated with the exclusion of the most variable MEP measurements. To this end, we selected 80% of the measurements with the lowest CVs of the individual MEP amplitudes. Each analysis was performed on the subset of subjects in which the required measurements were selected (the value at T1 was required in all cases for normalization). The resulting sample sizes ranged from 10 to 14 for different analyses. 

Among the effects of each protocol at every time point (MEP differences from the baseline), the protocol *iTBS 0* at T2 reached *p_unc_* = 0.03 (median MEP 113% of baseline), while others had *p_unc_* > 0.05. However, after a correction for multiple comparisons all differences became non-significant (*p_corr_* = 0.24 for *iTBS 0*).

In all the pairwise comparisons of the effects, *p*-values greater than 0.05 were obtained both in the Wilcoxon’s tests of mean MEPs and in the binomial tests comparing the rates of MEP facilitation and inhibition. In the Fisher’s tests of the association between the facilitation in the different conditions, uncorrected *p*-values smaller than 0.05 were obtained for the above-mentioned two pairs of conditions that had *p_unc_* < 0.05 in the whole-sample analysis, with both correlations being positive. The tests of the association of inhibition in different conditions produced *p_unc_* < 0.05 only for the positive correlation between T2 and T3 in the *iTBS 0-60* (*p_unc_* = 0.01). However, these results do not pass a correction for multiple comparisons. 

### 3.7. Analysis Excluding Subjects with Largest Variability of Baseline MEPs between Sessions

Similar analyses were performed with the exclusion of the subjects with the largest baseline MEP variability between sessions. For this, we selected 50% of the subjects (*n* = 8) having the lowest CVs of the mean MEPs at T1 in all four of the sessions.

All the effects of the protocols at every time point yielded *p* > 0.05. In all the pairwise comparisons of the effects, *p*-values greater than 0.05 were obtained both in the Wilcoxon’s tests comparing mean MEPs and in the binomial tests comparing the rates of MEP facilitation and inhibition. The tests of association between the MEP facilitation in different conditions produced *p* < 0.05 only for the correlation between T2 and T3 in the *iTBS 0-15* (*p_unc_* = 0.02), while the correlations of inhibition in different conditions produced *p_unc_* < 0.05 only for the positive correlation between T2 and T3 in the *iTBS 0-60* (*p_unc_* = 0.03). As in the analysis of the participants with the lowest within-session CVs of individual MEP amplitudes, these comparisons became non-significant when applying a correction for multiple comparisons (*p_corr_* = 0.24 for T2-T3 in *iTBS 0-15*; *p_corr_* = 0.36 for T2-T3 in *iTBS 0-60*).

### 3.8. Analysis of Effect Reliability

**Figure 5:**
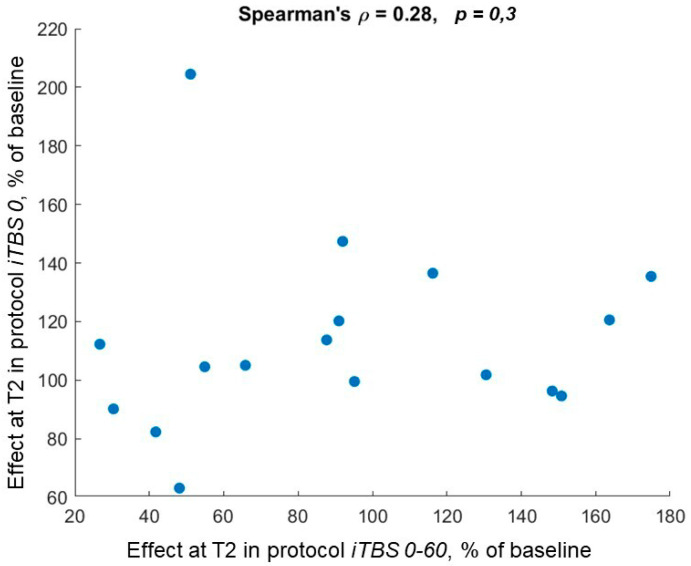
Between-session reliability of the effect of a single block of iTBS applied to M1 followed 15 min later by a block of iTBS of the vertex assessed by the correlation between the effects of *iTBS 0-60* and *iTBS 0* at T2.

A reliability analysis was performed for the effect of a single block of iTBS applied to M1, followed 15 min later by a block of iTBS of the vertex. This was implemented by computing the Spearman correlation between the effects at T2 in the protocols *iTBS 0-60* and *iTBS 0*, which yielded ρ = 0.28 (*p* = 0.3) (Figure 5). Thus, the data did not indicate that the individuals in which this TMS sequence increases MEPs on one day will respond similarly on another day. This is in line with the results obtained by the binarization of the data with respect to facilitation. Indeed, there were only 3 out of 17 subjects (18%) who were facilitators at T2 under both protocols *iTBS 0-60* and *iTBS 0*, and the facilitation events did not show a significant association (*p* = 1, Fisher’s exact test).

### 3.9. TBS Tolerability

The repeated TBS protocols used in our study were safe and, in general, well tolerated by healthy volunteers. No serious adverse events (AEs), such as seizures, occurred during the study. Only two cases of drop-out were caused by AEs that occurred within 24 h after stimulation: one case of intense headache (7 points of numerical pain rating scale (NPRS)), which began within 30 min after iTBS, resembled the headaches reported by the participant before participation in the study, and was resolved after the intake of a combined non-opioid analgesic, and one case of unpleasant feeling of pressure at the stimulation site, which resolved spontaneously. 

In the study, AEs occurred during 52 sessions (62.4% of the analyzed questionnaires) and after 15 sessions (27.3%) and were predominantly mild (93.4%). The most prevalent AEs during stimulation were sleepiness (38 sessions; 50%), non-painful scalp muscle contractions (23; 30.3%) or mild-to-moderate pain (14; 18.4%) at the stimulation site; within 24 h after stimulation—headaches (9 cases; 16.4% of the analyzed sessions); pain in the neck (3; 5.5%), mood changes (3; 5.5%), and transient tinnitus (3; 5.5%).

## 4. Discussion

In this study, we investigated the effects of the repeated iTBS protocols with 15 and 60 min intervals between blocks on motor cortex excitability measured by MEP amplitudes and the rates of the responders to iTBS. We also compared the effects of the repeated protocols with the effect of a single iTBS block and the stimulation of a functionally insignificant area. All the protocols used in our study were safe and well tolerated by the participants. We have shown that adding a second iTBS block did not significantly influence the MEP amplitude change and the responder rates, either with a 15 min or a 60 min interval between the active iTBS blocks. Moreover, there were no significant effects of both repeated protocols and a single iTBS block on the MEP amplitudes. Furthermore, in our study we observed low reliability for the responsiveness to a single active iTBS block between the two different stimulation days. 

It has been shown that a single iTBS block increases cortical excitability, probably by inducing long-term plasticity (LTP-like) changes [43]. However, the individual effects of a single iTBS block are highly variable: the reported rates of subjects showing the expected response to a single TBS block are less than 50% [13,32,44,45], which is consistent with the facilitator rate observed at T2 for *iTBS 0* in our study (47%). 

A way to increase TBS effectiveness is the use of repeated iTBS protocols, in which a second and further iTBS blocks are added to the first block. It has been shown that adding a second block separated by 10 min from the first one reduces the variability of the response to cTBS [42]. However, the effects of such protocols on the variability of the iTBS effects are poorly investigated. In this study, we have shown no significant differences of responder rates when adding a second M1 iTBS block, both 15 and 60 min after the first one, compared to adding vertex iTBS blocks with the same timing. Comparably to our study, Tse et al. (2018) found no significant effects of adding a second iTBS block, separated by both 5 and 15 min from the first block, on the responder rate [32]. It has also been shown that three blocks of iTBS separated by 15 min had no effect on the response to iTBS in the participants considered to be non-responders [39]. 

It has been suggested that the effects of repeated iTBS protocols might be influenced by the time interval between blocks because of the metaplasticity mechanisms [26,28]. However, the reported data regarding the dependence of iTBS effects on the time between separate blocks are inconsistent. In some studies, there were no significant neurophysiological effects of repeated iTBS protocols consisting of two blocks separated by an interval of 15 min [46] or 20 min [31], which is consistent with the results of our study, where no significant effects of *iTBS 0-15* at T2 were observed. In contrast to these results, there are studies showing significant differences between MEPs at the baseline and after two iTBS blocks separated by 10 [47] or 15 min [31]. One possible reason for such inconsistency might be the differences in iTBS protocols applied in these studies, e.g., Murakami et al. (2012) showed different effects of repeated iTBS protocols where active blocks were separated by 15 min when different stimulation intensities were applied (inhibition for priming iTBS with the intensity of 80% of active MT and no differences of MEP amplitude when a lower intensity of the priming protocol was applied (70% of active MT)) [31]. 

A particularly novel aspect of our study is the investigation of the effects of a repeated iTBS protocol with blocks separated by a prolonged interval (60 min). Our choice of the time delay was based on the proposal that such an interval might be optimal for the induction of additive metaplasticity, which was confirmed in animal studies (for a review see [33]). However, we have not observed an increase in the facilitatory effects of iTBS that could be expected if additive non-homeostatic metaplasticity mechanisms were to be induced using such a protocol, which is also inconsistent with the hypothesis of the “critical time window” for the induction of different metaplasticity types [28].

It should be noted that, in contrast to some previous studies investigating repeated iTBS protocols [31,46], in our study we have not found both significant effects of a single active iTBS block on MEP amplitudes and no differences between the *iTBS 0* and *Control* protocols. Therefore, it is possible that in our study the first block simply could not influence the effects of the second one regardless of the time interval between them because it had no effect on the synaptic plasticity itself. However, according to current knowledge about metaplasticity, it is induced by the influence on the threshold for different synaptic plasticity processes, which can occur even in the absence of significant synaptic plasticity after the first protocol. The results of a study in which low-intensity TBS priming protocols with no significant effects on MEP amplitudes increased the effects of single iTBS block confirm this hypothesis [31]. Moreover, in another study, a single iTBS block had no influence on MEP amplitudes, whereas repeated iTBS blocks and an cTBS–iTBS protocol led to significant MEP increases [47]. Of note, MEP amplitude changes are only an indirect measure of motor cortex excitability, and their absence does not fully exclude the effects of a single iTBS block on synaptic plasticity. Moreover, the high variability of the response to iTBS at the inter-individual level might partially explain the lack of a single iTBS block effect in some studies at the group level.

Because high inter-individual variability of iTBS effects might be the reason for the absence of significant effects at the group level, a possible way to increase its effectiveness could be the investigation of possible predictors of individual response to stimulation [18]. However, a low reliability of individual response to iTBS has been confirmed recently by Boucher et al. (2021), in which only 8% of the participants showed the expected facilitatory response in different TBS sessions [14]. To investigate the reliability of the response to TBS in different sessions, we calculated the correlation between the responder classifications after the first block of active iTBS, followed by vertex stimulation in the *iTBS 0* and *iTBS 0-60* protocols. According to our hypothesis, the MEPs at this time point (T2) reflect the effect of a single active iTBS block, whose duration is approximately 20 min [43]. However, we have observed no significant correlation, which is also consistent with the previously published data showing that the effects of iTBS on MEP magnitude were not reproducible at both the group and individual level between two visits separated by approximately 7 days [48]. Similar findings were observed by Perellon-Alonso et al. (2018), showing no significant reliability of response to either active or sham iTBS applied over 5 consecutive days and a low likelihood to remain a responder between consecutive days [49]. Thus, according to these results, the binarization of responders and non-responders to iTBS might be of a low value, at least for its neurophysiological effects in healthy persons.

It should also be noted that there was a significant correlation between the events of inhibition after one block of M1 iTBS followed by one block of vertex iTBS with the events of inhibition after the same sequence followed by an additional block of M1 iTBS 60 min after the first block (inhibition at time points T2 and T3 in protocol *iTBS 0-60*). Because it has been shown that the effect of a single iTBS block lasts approximately 20 min [43] and no significant differences between *iTBS 0-60* and *iTBS 0* were observed on the MEP ratio, confirming no metaplasticity effects, this correlation can reflect the effects of a single iTBS block. Thus, we suggest that it could be explained by the short-term reproducibility of the iTBS effect on motor cortex excitability. Therefore, considering that there were no significant correlations between responses to iTBS when they were assessed on different visits separated by several days, it can be hypothesized that responsiveness to iTBS is a short-term rather than a constant condition. However, it should be mentioned that the time used to assess the effect of a single iTBS block was different in this comparison: 15 min when assessing the effect of the first block and immediately after iTBS when assessing the effect of the second active iTBS block. Moreover, fluctuations in MEP amplitude and not the effect of iTBS can also explain the observed correlation. Therefore, further studies are needed to investigate the time-dependency of the responsiveness to iTBS, assessing both the short- and long-term variability of its effects and accounting for MEP fluctuations.

Possible reasons for the low responder rates in the studies investigating the effects of the TBS protocols on primary motor cortex excitability include not only the variability of the TBS effects per se, but also the high variability of the MEPs as a measure of neurophysiological response [3,45]. This variability can be explained by both the limitations of TMS accuracy (e.g., variability of coil placement) and the complex neurophysiological nature of the TMS-induced response of the stimulated M1 area. The first source of variability was minimized in our study by using a robotic neuronavigation system to accurately determine and maintain the position of the stimulating coil. Moreover, in our study, the removal of participants with the most variable MEPs (considering either the within-session MEP variability or the between-session fluctuations of the baseline mean MEP values) has not substantially influenced the results of both the within- and the between-protocol comparisons of MEP ratios and responder rates. Our data also provide no evidence that session-to-session fluctuations in the initial M1 excitability (as measured by the mean MEP at T1) produce a consistent influence on the effect on the MEPs of a single block of M1 iTBS (followed by vertex iTBS). Therefore, the variability of the MEPs could only partially explain the low reproducibility of the iTBS effects observed in our study. Our data are in line with the results of a previously published study showing no correlation between MEP reproducibility and the response to iTBS assessed as MEP modulation in healthy volunteers receiving either active or sham iTBS over 5 consecutive days [49].

In our study, we also have not found any association of the stimulation effect with the baseline MEP variability that could pass a correction for multiple comparisons. However, it should be noted that all the correlation coefficients were negative. In the case of statistical significance, this would show an association between higher MEP variability and a reduced response to iTBS, while a reverse association was shown in the study by Hordacre et al. (2016) for the cTBS protocol [50]. Thus, different effects of MEP variability on iTBS and cTBS protocols could be hypothesized; however, further studies are needed in order to test this assumption.

Other measures of neurophysiological effects, e.g., TMS-evoked potentials (TEPs), which are more reproducible than MEPs [51,52,53] could be potentially helpful in investigating the TBS modulatory effects. However, according to a recently published study, the individual effects of a single iTBS or cTBS block on TEPs were also not reproduced between separate visits [54]. Another way to assess TBS effects and their variability is to use not neurophysiological, but behavioral or clinical measures. Although preliminary data show a low reproducibility of a single iTBS block effect on the performance of a response inhibition task [55], further investigations are needed to determine whether repeated iTBS protocols influence the response to iTBS assessed by motor or cognitive tasks in a way similar to the neurophysiological effects of these protocols.

Another possible way to increase the effects of repeated iTBS protocols might be to increase the number of blocks of active iTBS. Nettekoven et al. (2014) reported a significant effect of three, but not two, iTBS blocks on motor cortex excitability [38]. Moreover, there are studies showing the clinical effectiveness of accelerated iTBS protocols including multiple stimulation blocks, e.g., Stanford Neuromodulation therapy [30,56]. Therefore, the absence of the significant effects of repeated protocols consisting of two active iTBS blocks observed in our study does not imply the ineffectiveness of accelerated protocols including more iTBS blocks. 

### Limitations

The limitations of this study include a small sample size (especially in the additional analyses excluding participants with high MEP variability) and a relatively small number of MEPs recorded at each time point. We also measured TBS effects only 15 and 60 min after the first TBS block. Therefore, we could have missed possible delayed neurophysiological effects of the repeated protocols, which should be investigated in further studies. Moreover, we used differences between MEPs at various time points for some comparisons, e.g., T2-T1 and T3-T2 to compare the responses to a single iTBS block at different visits; however, both these intervals fall within the time window of the proposed duration of the iTBS effect [43]. 

It should also be noted that we used iTBS of the vertex and not a sham TMS as a control condition and have not assessed the participants’ awareness of the stimulation protocol they received, which could contribute to the stimulation effects. However, considering the primary aim of our study, the success of blinding was supported by the fact that the participants were not aware of the effects of different control and active block stimulation combinations even when they could potentially discriminate that stimulation was applied to different sites. Furthermore, the stimulation of the vertex is widely used as a control in other studies investigating repeated iTBS protocols (e.g., [38,39]). 

In conclusion, it should be mentioned that the non-significant results regarding both the neurophysiological effects and the rates of the responders to the stimulation observed in our study should be interpreted only as the neurophysiological ineffectiveness of repeated protocols with the parameters described in our study when applying them to M1. Furthermore, the results obtained from the studies assessing the variability of the iTBS neurophysiological effects between healthy volunteers cannot be directly translated into clinical practice. Further investigations are needed in order to continue the collection of data regarding the influence of different repeated protocols, not only on MEPs, but also on other neurophysiological measures (e.g., TEPs) or their behavioral and clinical effects, as well as the variability of the response to these protocols.

## Figures and Tables

**Table 1:** Effects of the single protocols on the mean MEP amplitudes at time points T2 and T3.

Protocol	Median MEP Ratio to Baseline, %		Uncorrected *p*-Value for MEP Ratio Comparison with 100%		Bonferroni-Corrected *p*-Value		Median Difference of MEP Ratio between T2 and T3, %	Uncorrected *p*-Value for MEP Ratio Comparisons between T2 and T3	Bonferroni-Corrected *p*-Value
	T2	T3	T2	T3	T2	T3			
*iTBS 0-15* ^1^	96.7	98.3	0.98 ^2^	0.79	1.0	1.0	3.6	0.59	1.0
*iTBS 0-60*	91	87.2	0.49	0.27	1.0	1.0	6.8	0.33	1.0
*iTBS 0*	105	90.2	0.1	0.25	1.0	1.0	14.1	0.011	0.12
*Control*	95.9	96.4	0.62	0.75	1.0	1.0	2.5	0.91	1.0

^1^ Protocol abbreviations: *iTBS 0-15*—repeated iTBS protocol with 2 active blocks with 15 min between them and a control block 60 min after the first active block; *iTBS 0-60*—repeated iTBS protocol with an active block, a control stimulation block 15 min after it, and a second active iTBS block 60 min after the first active block; *iTBS 0*—an active iTBS block followed by 2 control stimulation blocks 15 and 60 min after it; *Control*—3 control stimulation blocks (baseline, 15, and 60 min after the first block); ^2^ *p_T2_* and *p_T3_*—*p*-values for comparisons at T2 and T3 with the baseline, respectively (Wilcoxon’s signed-rank test).

**Table 2:** Comparison of protocol effects on MEP ratio and rates of responders at time points T2 and T3.

Comparison	Median Difference of MEP Ratio, %		*p*-Value for Difference of MEP Ratio with 100%		Difference of Facilitator Rates		*p*-Value for Facilitation Rate Comparison between T2 and T3		Difference of Inhibitor Rates		*p*-Value for Inhibition Rate Comparison between T2 and T3	
	T2	T3	Uncorrected	Bonferroni-Corrected	T2	T3	Uncorrected	Bonferroni-Corrected	T2	T3	Uncorrected	Bonferroni-Corrected
*iTBS 0-15* vs. *iTBS 0-60*	5.8	1	*p_T2_* = 0.21 ^1^ *p_T3_* = 0.46	*p_T2_* = 1.0 *p_T3_* =1.0	−6	0	*p_T2_* = 1 *p_T3_* = 1	*p_T2_* = 1.0 *p_T3_* =1.0	−12	−12	*p_T2_* = 0.69 *p_T3_* = 0.73	*p_T2_* = 1.0 *p_T3_* =1.0
*iTBS 0-15* vs. *iTBS 0*	−20.3	−0.3	*p_T2_* = 0.76 *p_T3_* = 0.62	*p_T2_* = 1.0 *p_T3_* = 1.0	−18	0	*p_T2_* = 0.55 *p_T3_* = 1	*p_T2_* = 1.0 *p_T3_* = 1.0	23	−6	*p_T2_* = 0.22 *p_T3_* = 1	*p_T2_* = 1.0 *p_T3_* = 1.0
*iTBS 0-15* vs. *Control*	−19.2	2.3	*p_T2_* = 0.83 *p_T3_* = 0.98	*p_T2_* = 1.0 *p_T3_* = 1.0	5	−17	*p_T2_* = 1 *p_T3_* = 0.45	*p_T2_* = 1.0 *p_T3_* = 1.0	−6	−6	*p_T2_* = 1 *p_T3_* = 1	*p_T2_* = 1.0 *p_T3_* = 1.0
*iTBS 0-60* vs. *iTBS 0*	−26	−5.5	*p_T2_* = 0.19 *p_T3_* = 0.91	*p_T2_* = 1.0 *p_T3_* = 1.0	−12	0	*p_T2_* = 1 *p_T3_* = 0.22	*p_T2_* = 1.0 *p_T3_* = 1.0	35	6	*p_T2_* = 0.031 *p_T3_* = 1	*p_T2_* = 0.36 *p_T3_* = 1.0
*iTBS 0-60* vs. *Control*	−16.4	−17.1	*p_T2_* = 0.38 *p_T3_* = 0.38	*p_T2_* = 1.0 *p_T3_* = 1.0	11	−17	*p_T2_* = 0.69 *p_T3_* = 0.51	*p_T2_* = 1.0 *p_T3_* = 1.0	6	6	*p_T2_* = 1 *p_T3_* = 1	*p_T2_* = 1.0 *p_T3_* = 1.0
*iTBS 0* vs. *Control*	1.4	−10.4	*p_T2_* = 0.52 *p_T3_* = 0.55	*p_T2_* = 1.0 *p_T3_* = 1.0	23	−17	*p_T2_* = 0.22 *p_T3_* = 0.45	*p_T2_* = 1.0 *p_T3_* = 1.0	−29	0	*p_T2_* = 0.063 *p_T3_* = 1	*p_T2_* = 0.76 *p_T3_* = 1.0

^1^ *p_T2_* and *p_T3_*—*p*-values for comparisons at T2 and T3, respectively (Wilcoxon’s signed-rank test for comparisons of effects on mean MEP ratio and binomial test for comparisons of facilitator or inhibitor rates).

**Table 3:** Effects of single protocols on the responder rates at time points T2 and T3.

Protocol	Rate of Facilitators, %		Uncorrected *p*-Value for Facilitation Rate Comparison between T2 and T3	Bonferroni-Corrected *p*-Value	Rate of Inhibitors, %		Uncorrected *p*-Value for Inhibition Rate Comparison between T2 and T3	Bonferroni-Corrected *p*-Value
	T2	T3			T2	T3		
*iTBS 0-15*	29	24	1.0	1.0	35	41	1.0	1.0
*iTBS 0-60*	35	24	0.5	1.0	47	53	1.0	1.0
*iTBS 0*	47	24	0.22	1.0	12	47	0.031	1.0
*Control*	24	41	0.45	1.0	41	47	1.0	1.0

**Table 4:** Correlations between responder classifications with *p* < 0.05 before correction for multiple comparisons (exact Fisher’s test).

Facilitation			
	Facilitation in *iTBS 0-60* at T3	No Facilitation in *iTBS 0-60* at T3	Odds Ratio
Facilitation in *iTBS 0-60* at T2	4/17	2/17	inf. ^1^
No facilitation in *iTBS 0-60* at T2	0/17	11/17	
	Facilitation in *iTBS 0-60* at T3	No facilitation in *iTBS 0-60* at T3	
Facilitation in *iTBS 0-15* at T3	3/17	1/17	36
No facilitation in *iTBS 0-15* at T3	1/17	12/17	
Inhibition			
	Inhibition in *iTBS 0-15* at T3	No Inhibition in *iTBS 0-15* at T3	Odds Ratio
Inhibition in *iTBS 0-15* at T2	5/17	1/17	23
No inhibition in *iTBS 0-15* at T2	2/17	9/17	
	Inhibition in *iTBS 0-60* at T3	No inhibition in *iTBS 0-60* at T3	
Inhibition in *iTBS 0-60* at T2	8/17	0/17	inf.
No inhibition in *iTBS 0-60* at T2	1/17	8/17	

^1^ inf.—an infinite value (due to an entry in the contingency table being zero).

## Data Availability

The data presented in this study are available on a reasonable request from the corresponding author.

## Supplementary Materials

The following supporting information can be downloaded at: https://www.mdpi.com/article/10.3390/brainsci12081064/s1, Figure S1: individual plots of MEP amplitudes at each time point for the protocol *iTBS 0-15*, Figure S2: individual plots of MEP amplitudes at each time point for the protocol *iTBS 0-60*, Figure S3: individual plots of MEP amplitudes at each time point for the protocol *iTBS 0*, Figure S4: individual plots of MEP amplitudes at each time point for the protocol *Control*.
