# Supplementary material for: Adding a Second iTBS Block in 15 or 60 Min Time Interval Does Not Increase iTBS Effects on Motor Cortex Excitability and the Responder Rates
Source: Brain Sci. 2022 Aug 11;12(8):1064. doi: 10.3390/brainsci12081064 (PMC9405900; doi:10.3390/brainsci12081064)
Supplement: Supplementary file 1 [file brainsci-12-01064-s001.zip › brainsci-1838967-supplementary.pdf]

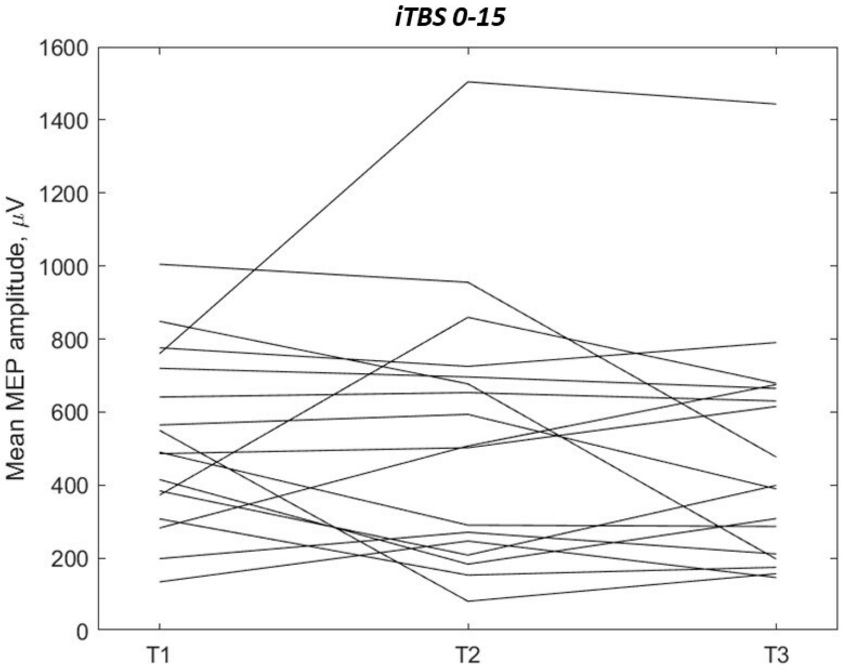

Figure S1. individual plots of MEP amplitudes at each time point for the protocol *iTBS 0-15*.

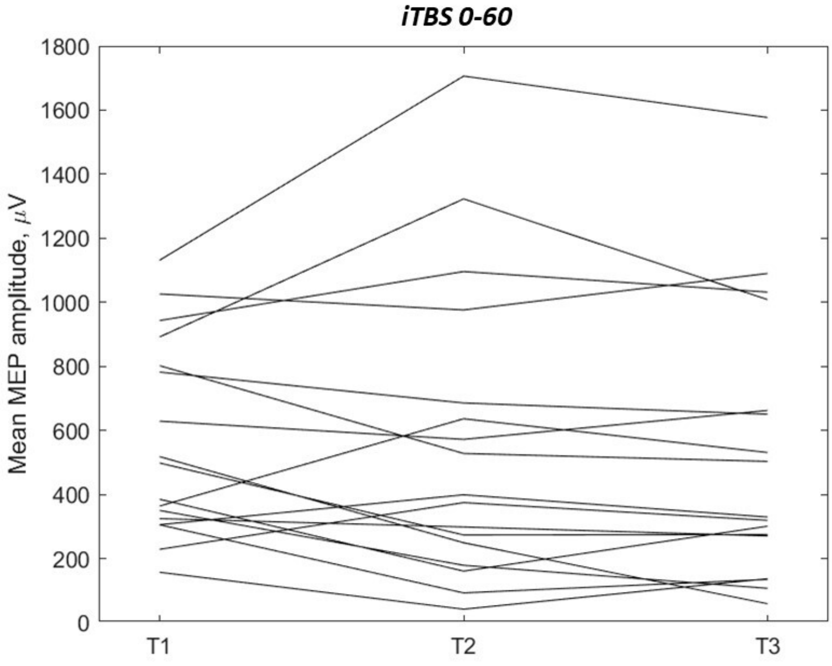

Figure S2. individual plots of MEP amplitudes at each time point for the protocol *iTBS 0-60*.

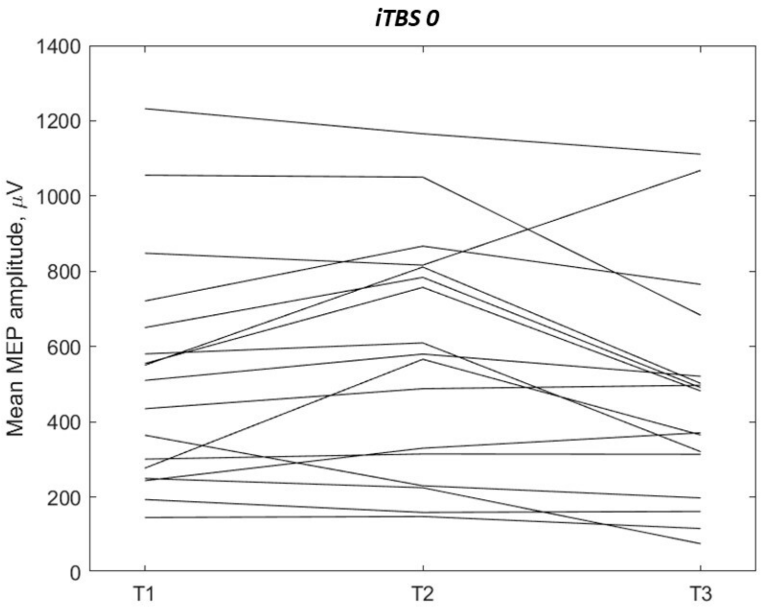

Figure S3. individual plots of MEP amplitudes at each time point for the protocol *iTBS 0*.

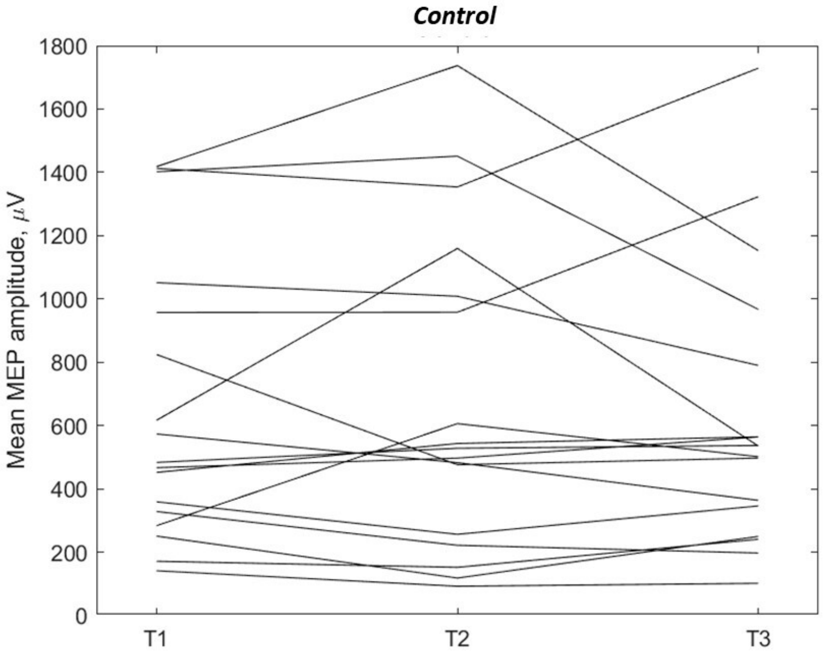

Figure S4. individual plots of MEP amplitudes at each time point for the protocol *Control*.
